# Scoliosis instrumentation alters primary and coupled motions of the spine: An in vitro study using entire thoracolumbar spine and rib cage specimens

**DOI:** 10.1002/jsp2.70028

**Published:** 2024-12-18

**Authors:** Christian Liebsch, Peter Obid, Morten Vogt, Benedikt Schlager, Hans‐Joachim Wilke

**Affiliations:** ^1^ Institute of Orthopaedic Research and Biomechanics, Trauma Research Centre Ulm, Ulm University Medical Centre Ulm Germany; ^2^ Department of Orthopaedics and Trauma Surgery Freiburg University Medical Centre Freiburg Germany

**Keywords:** adolescent idiopathic scoliosis, biomechanics, coupled motions, in vitro study, lumbar spine, posterior instrumentation, range of motion, rib cage, thoracic spine

## Abstract

**Background:**

Effects of rigid posterior instrumentation on the three‐dimensional post‐operative spinal flexibility are widely unknown. Purpose of this in vitro study was to quantify these effects for characteristic adolescent idiopathic scoliosis instrumentations.

**Methods:**

Six fresh frozen human thoracic and lumbar spine specimens (C7‐S) with entire rib cage from young adult donors (26–45 years) without clinically relevant deformity were loaded quasi‐statically with pure moments of 5 Nm in flexion/extension, lateral bending, and axial rotation. Primary and coupled motions of all segments were measured using optical motion tracking. Specimens were tested without instrumentation and with posterior rod instrumentations ranging from T2 to L1 (for Lenke Type 2) and from T8 to L3 (for Lenke Type 5) based on survey results among spinal deformity surgeons. Statistical differences were evaluated using the pairwise Friedman test.

**Results:**

Primary ranges of motion were significantly (*p* < 0.05) reduced in all six motion directions in the entire thoracic spine (T1‐L1) for both instrumentations, but solely in extension and axial rotation in the entire lumbar spine (L1‐S) for T8‐L3 instrumentation. Without instrumentation, strong ipsilateral axial rotation during primary lateral bending and strong contralateral lateral bending during primary axial rotation were detected in the thoracic spine (T1‐L1) and slight inverse coupled motions in the lumbar spine (L1‐S). While coupled axial rotation was significantly (*p* < 0.05) reduced, especially in the upper thoracic spine (T1‐T5) for T2‐L1 instrumentation and in the lumbar spine (L1‐S) for T8‐L3 instrumentation, coupled lateral bending was solely significantly (*p* < 0.05) reduced in the upper thoracic spine (T1‐T5) for T2‐L1 instrumentation. Coupled motions in primary flexion and extension were non‐existent and not affected by any fixation (*p* > 0.05).

**Conclusions:**

Instrumentation reduces the primary flexibility and diminishes the natural coupling behavior between lateral bending and axial rotation, primarily in the upper thoracic spine, potentially causing correction loss and junctional deformity in the long‐term.

## Graphical abstract

Six human thoracic and lumbar spine specimens (C7‐S) with entire rib cage from young adult donors (26–45 years) without clinically relevant deformity were biomechanically tested to investigate primary and coupled motions of all segments without instrumentation and with posterior rod instrumentations ranging from T2 to L1 and from T8 to L3. Instrumentation was found to significantly (*p* < 0.05) reduce the primary flexibility in all motion directions and to diminish the natural coupling behavior between lateral bending and axial rotation, primarily in the upper thoracic spine, potentially causing correction loss and junctional deformity in the long‐term.

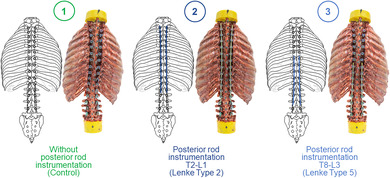

## 1 INTRODUCTION

Adolescent idiopathic scoliosis (AIS) is a three‐dimensional spinal deformity with the principal characteristic of unphysiologically high curvature in the frontal plane, requiring corrective surgery in severe cases. While surgery including posterior screw‐rod instrumentation represents a common procedure for the treatment of AIS patients, effects of such rigid spinal instrumentation on the three‐dimensional motion behavior are still indeterminate, though potentially affecting the patients' movement comfort and the long‐term treatment outcome in terms of the commonly diagnosed three‐dimensional post‐operative correction loss.^1^, ^2^ Based on qualitative in vivo and in vitro studies in the early 20th century, Lovett already suggested a role of coupled motions of the thoracic and lumbar spine in the development and progression of idiopathic scoliosis.^3^, ^4^ Coupled motions, also known as secondary or out‐of‐plane motions, appear intrinsically during spinal movements in the primary motion plane. Findings of more recent in vivo studies clearly show that the coupled motion behavior is considerably altered in the scoliotic spine when compared to the healthy, non‐deformed spine,^5^, ^6^, ^7^ while the latter exhibits a strong and consistent motion coupling between lateral bending and axial rotation, particularly in the thoracic spine.^8^, ^9^, ^10^ However, in vivo studies generally entail limitations regarding the detailed assessability of coupled motions due to the individual, heterogeneous movement comfort of the participants as well as imprecise measuring techniques. To fully understand potential effects of AIS instrumentation on the post‐operative three‐dimensional motion behavior, in vitro as well as in silico studies under standardized and reproducible testing conditions are thus essential. The purpose of this in vitro study therefore was to explore potential effects of common AIS instrumentation configurations on the primary and coupled motions of the entire thoracic and lumbar spine.

## 2 MATERIALS AND METHODS

### 2.1 Specimens

Six fresh frozen human thoracic spine specimens (C7‐sacrum) with entire rib cages were prepared for experimental testing. The specimens originated from young adult donors (26–45 years, two female and four male) and were acquired from an accredited tissue bank (Science Care Inc., Phoenix, AZ, USA). Use of the specimens for this study was approved by the ethics committee of the University of Ulm (no. 63/17) and conforms with the Declaration of Helsinki. Prior to preparation, absence of any injuries, degenerative changes, and clinically relevant deformities was verified and adequate bone quality was ensured (BMD 80–164 mg/cc HA) using QCT scans (Siemens Somatom Definition AS, Siemens Healthcare, Erlangen, Germany). Any fat, nerve, and muscle tissue except for the intercostal muscles was removed during preparation, leaving all biomechanically relevant tissue including bone, cartilage, and ligaments intact. The upper‐ and lowermost vertebrae (C7 and sacrum, respectively) were embedded coaxially and parallelly to their endplates in polymethylmethacrylate (PMMA, Technovit 3040, Heraeus Kulzer, Wehrheim, Germany) in order to attach metal flanges for mounting in the testing device. The specimens were stored frozen at −20°C before and after preparation and testing and were thawed for about 12 h at 5°C prior to preparation and testing, which were performed at room temperature within a total of 20 h to prevent specimen decomposition.^11^ Additionally, the specimens were kept moist using 0.9% saline solution.

### 2.2 Experimental setup

**FIGURE 1:**
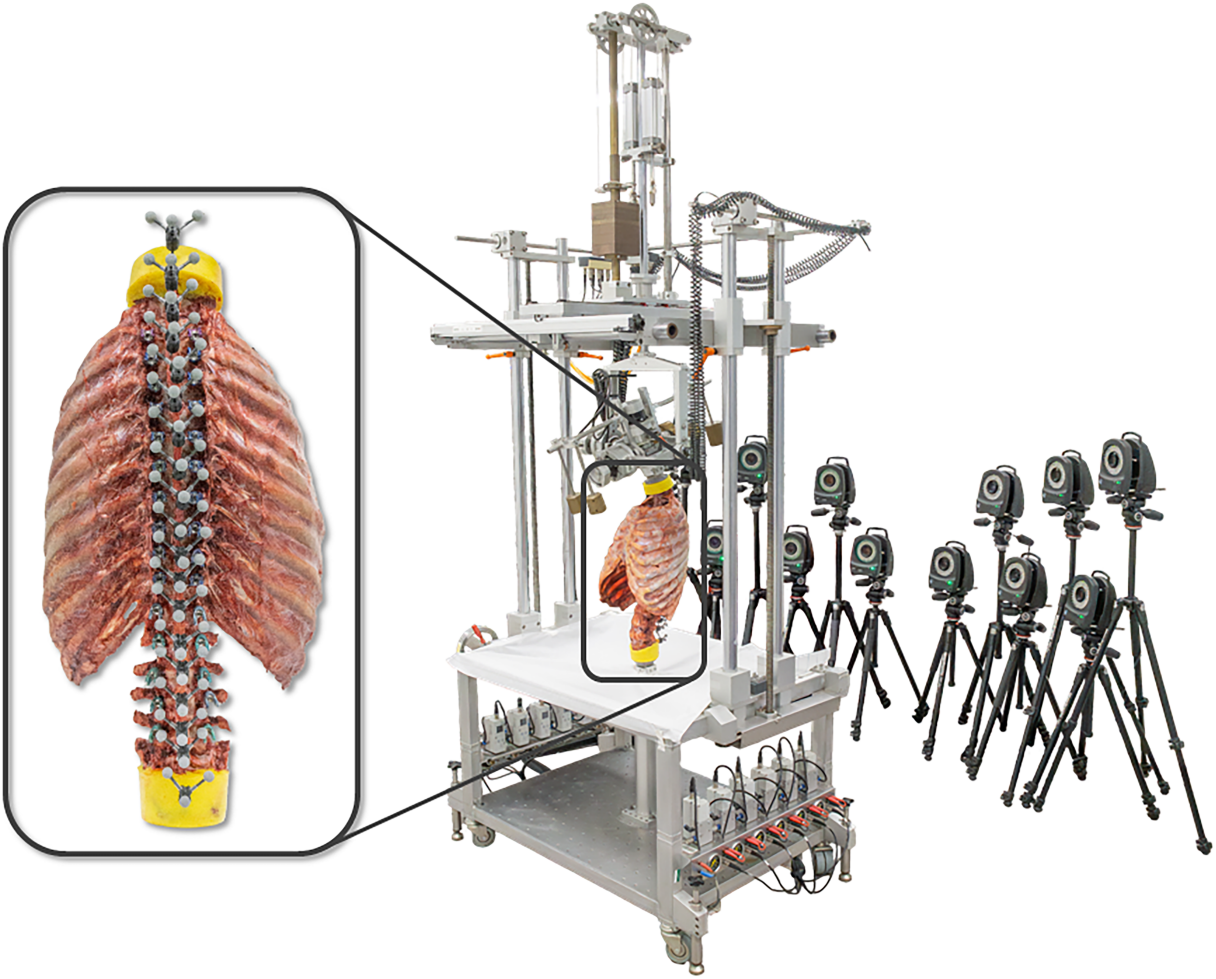
Overview of the experimental setup including the spine tester (middle), the optical motion tracking system consisting of 12 infra‐red cameras (right), and one of *n* = 6 thoracic and lumbar spine specimens (C7‐sacrum) with rib cage equipped with three retroreflective markers at each vertebral level (left).

The specimens were loaded quasi‐statically and displacement‐controlled (1°/s) with pure moments of 5 Nm^11^ in primary flexion/extension, lateral bending, and axial rotation using a well‐established spine tester^12^ (Figure 1 middle). Loading was performed for 3.5 cycles, of which the third full cycle was used for data evaluation, in order to reduce visco‐elastic effects.^11^ Relative motions of all segmental levels (C7‐sacrum) were captured during spinal loading using an optical motion tracking system (Vicon MX13+, Vicon Motion Systems Ltd., Oxford, UK) consisting of 12 free‐standing infra‐red cameras (Figure 1 right) in order to determine primary range of motion and coupled motions of each spinal motion segment. For this, each vertebra was equipped with three retroreflective markers (Figure 1 left). Load and displacement data were recorded with a sampling frequency of 50 Hz.

### 2.3 Testing conditions

**FIGURE 2:**
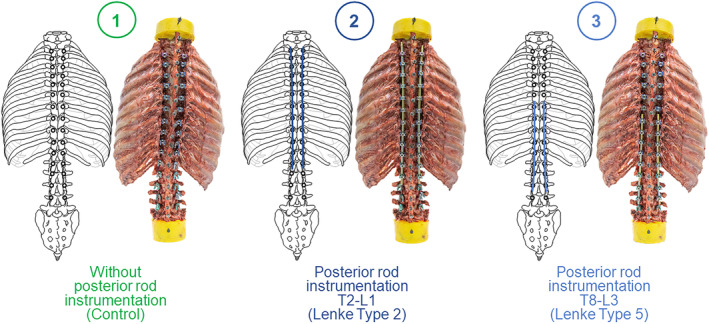
Overview of the testing conditions including the reference condition without posterior rod instrumentation (1), the condition with posterior rod instrumentation from T2 to L1 for Lenke Type 2 curve correction (2), and the condition with posterior rod instrumentation from T8 to L3 for Lenke Type 5 curve correction (3).

Experimental testing was performed in three steps (Figure 2): First, the specimens were tested without posterior rod instrumentation but with polyaxial pedicle screws inserted (Ennovate®, Aesculap AG, Tuttlingen, Germany), serving as control for the following two testing conditions. Screw sizes were selected by an experienced spine surgeon according to individual pedicle volumes and vertebra lengths, ranging from 4.5 × 30 mm to 4.5 × 40 mm at T2 level and from 6.5 × 50 mm to 7.5 × 50 mm at L3 level. In the second and third steps, the specimens were tested with posterior titanium rod instrumentation with a rod diameter of 5.5 mm (Ennovate®, Aesculap AG, Tuttlingen, Germany) from T2 to L1 (second condition) and from T8 to L3 (third condition) (Figure 2). Instrumentation lengths in terms of upper and lower instrumented vertebrae were based on survey results among AIS surgeons regarding their preferred surgical treatment^13^, ^14^ of Lenke curve types 2 (double thoracic curve) and 5 (thoraco−/lumbar curve).^15^

### 2.4 Data analysis and statistical evaluation

Load and displacement data were post‐processed using Excel 2019 (Microsoft Corp., Redmond, USA). Intersegmental primary and coupled motions of specific anatomical spinal sections were determined using a custom‐written Matlab script (Matlab R2021a, MathWorks Inc., Natick, USA). Statistical differences between the condition without posterior rod instrumentation and each condition with posterior rod instrumentation were evaluated using the pairwise Friedman test in SPSS27 (IBM Corp., Armonk, USA) with a significance level of 0.05.

## 3 RESULTS

### 3.1 Thoracic and lumbar spine (T1‐sacrum)

**FIGURE 3:**
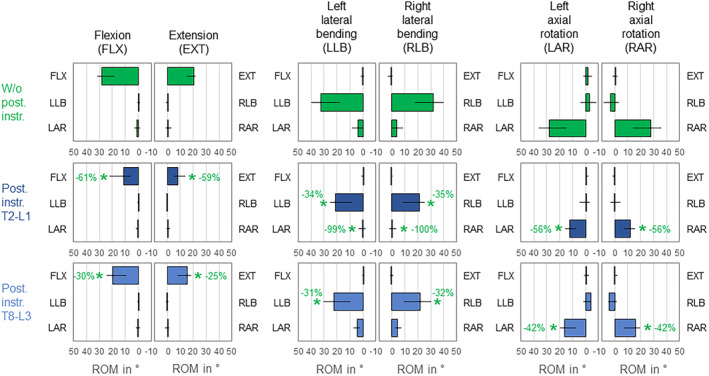
Effects of posterior rod instrumentation on primary and coupled motions of the overall thoracic and lumbar spine (T1‐sacrum). Data is represented as medians with minimum and maximum values (*n* = 6). Statistical differences (*p* < 0.05) compared to the condition without posterior rod instrumentation are marked with *.

The overall thoracic and lumbar spinal primary range of motion was significantly (*p* < 0.05) decreased by both instrumentation types in all six motion directions (Figure 3). Highest primary range of motion decreases were found for T2‐L1 instrumentation in flexion (−61%). Without posterior rod instrumentation, no considerable coupled motions were detected during primary flexion/extension, while in lateral bending, slight ipsilateral axial rotation, and in axial rotation, a slight tendency towards contralateral lateral bending were identified. Whereas T2‐L1 instrumentation led to significant (*p* < 0.05) and complete reduction of ipsilateral axial rotation during primary lateral bending in both directions (−99%/−100%), this coupled motion behavior was maintained with T8‐L3 instrumentation (*p* > 0.05). Similar findings were obtained in primary axial rotation, while the reduction of contralateral lateral bending was not statistically significant (*p* > 0.05). During primary flexion/extension, no significant changes of coupled motions were found (*p* > 0.05).

### 3.2 Thoracic spine (T1‐L1)

**FIGURE 4:**
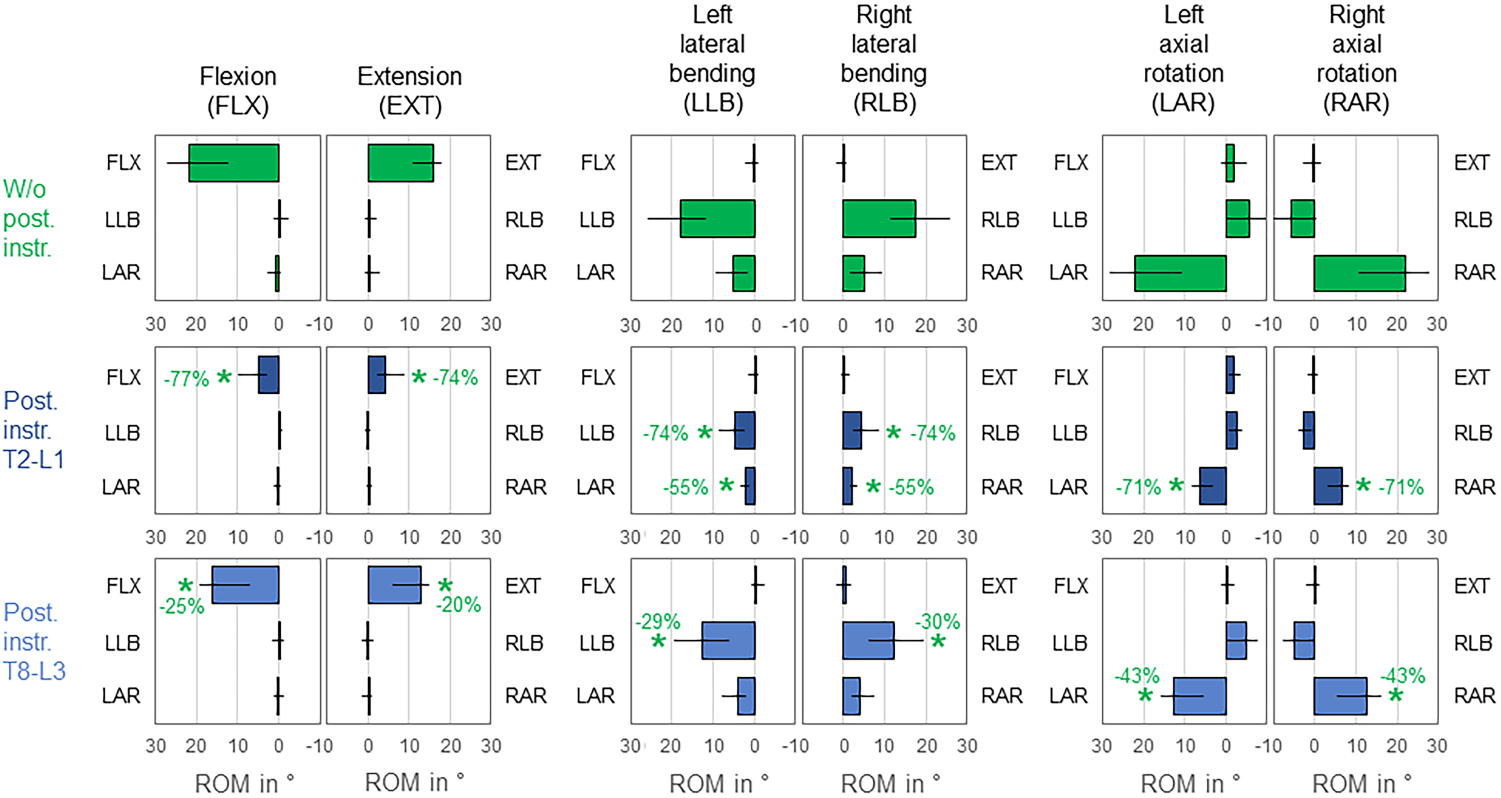
Effects of posterior rod instrumentation on primary and coupled motions of the overall thoracic spine (T1‐L1). Data is represented as medians with minimum and maximum values (*n* = 6). Statistical differences (*p* < 0.05) compared to the condition without posterior rod instrumentation are marked with *.

Both instrumentation types caused significant (*p* < 0.05) primary range of motion decreases of the overall thoracic spine in all six motion directions, while the highest reduction was found for T2‐L1 instrumentation in primary flexion (−77%, Figure 4). Considerable coupled motions were found in both primary lateral bending with ipsilateral axial rotation and primary axial rotation with contralateral lateral bending in the condition without posterior rod instrumentation, but not in primary flexion/extension. While T2‐L1 instrumentation led to significant (*p* < 0.05) reduction of coupled axial rotation during primary lateral bending in both directions (−55%/−55%), the coupled motion characteristics remained unaffected by posterior rod instrumentation in all other motion directions (*p* > 0.05).

### 3.3 Lumbar spine (L1‐sacrum)

**FIGURE 5:**
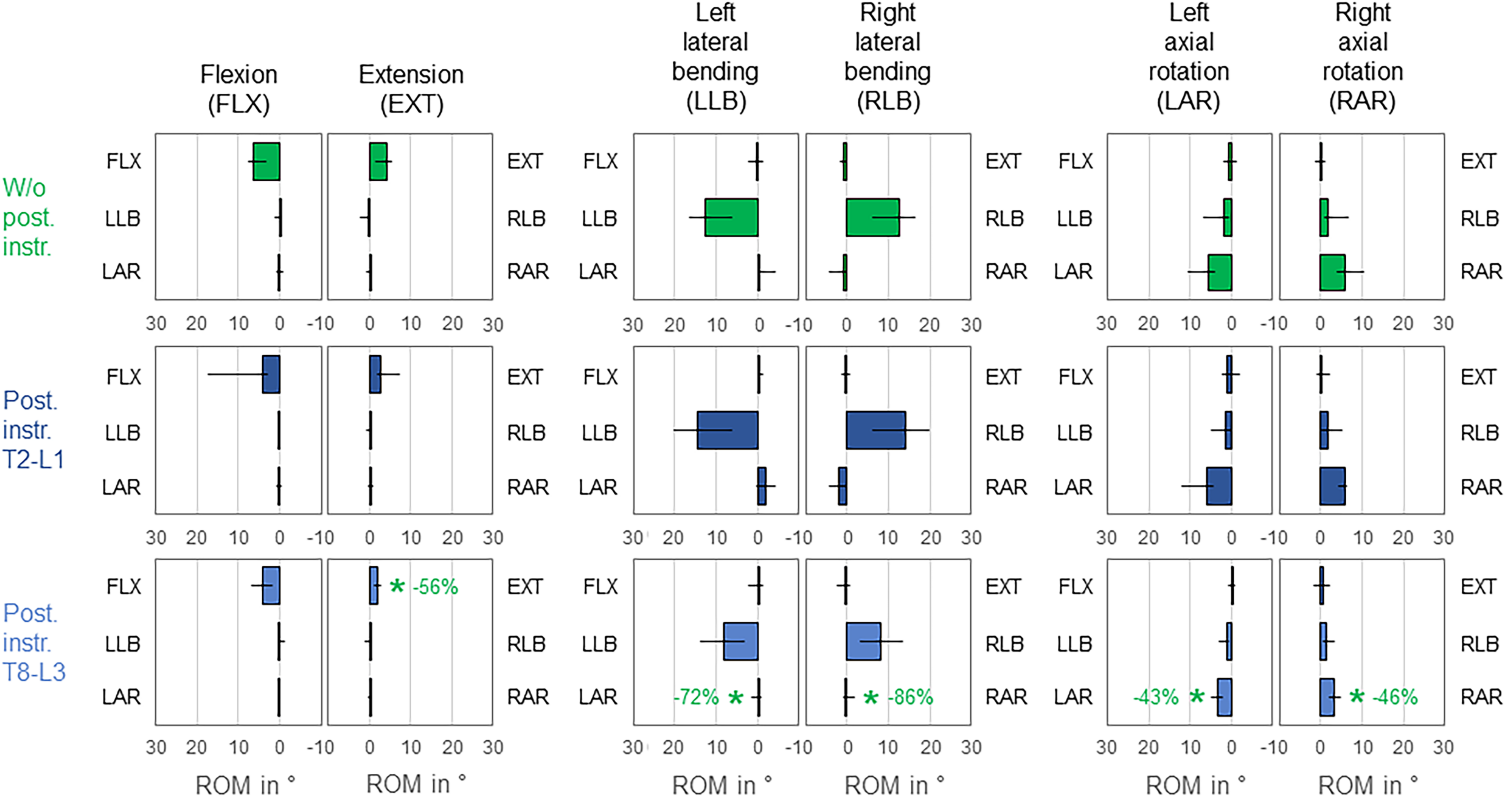
Effects of posterior rod instrumentation on primary and coupled motions of the overall lumbar spine (L1‐sacrum). Data is represented as medians with minimum and maximum values (*n* = 6). Statistical differences (*p* < 0.05) compared to the condition without posterior rod instrumentation are marked with *.

Primary range of motion of the overall lumbar spine was solely affected by T8‐L3 instrumentation, which led to significant (*p* < 0.05) range of motion reduction in primary extension, where the highest decrease was detected (−56%), as well as in primary left and right axial rotation (Figure 5). While no considerable coupled motions were identified during primary flexion/extension, slight contralateral axial rotation during primary lateral bending and ipsilateral lateral bending during primary axial rotation were detected in the condition without posterior rod instrumentation. T8‐L3 instrumentation caused significant (*p* < 0.05) and high reduction of contralateral axial rotation during primary lateral bending in both directions (−72%/−86%), whereas coupled lateral bending during primary axial rotation was not affected by either instrumentation type (*p* > 0.05).

### 3.4 Upper (T1‐T5), mid‐ (T5‐T9), and lower (T9‐L1) thoracic spine

**FIGURE 6:**
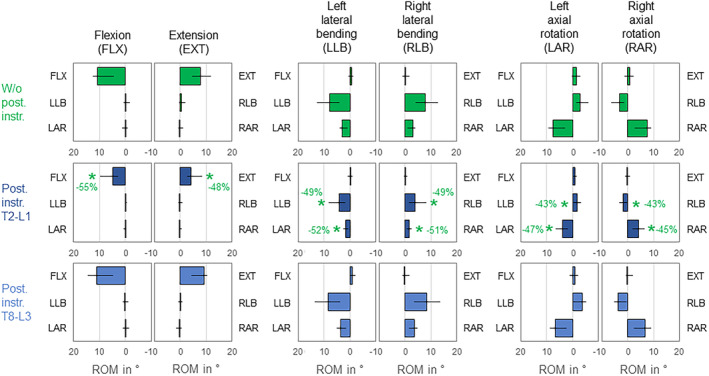
Effects of posterior rod instrumentation on primary and coupled motions of the upper third of the thoracic spine (T1‐T5). Data is represented as medians with minimum and maximum values (*n* = 6). Statistical differences (*p* < 0.05) compared to the condition without posterior rod instrumentation are marked with *.

The upper third of the thoracic spine was solely affected by T2‐L1 instrumentation, which caused significant (*p* < 0.05) primary range of motion reduction in all six motion directions (Figure 6). Highest decreases of primary range of motion due to T2‐L1 instrumentation were detected in flexion (−55%). Considerable coupled motions were found in the condition without posterior rod instrumentation with ipsilateral axial rotation during primary lateral bending as well as contralateral lateral bending during primary axial rotation, which were all significantly (*p* < 0.05) and highly decreased by T2‐L1 instrumentation (−52%/−51% in left/right lateral bending and −43%/−43% in left/right axial rotation).

**FIGURE 7:**
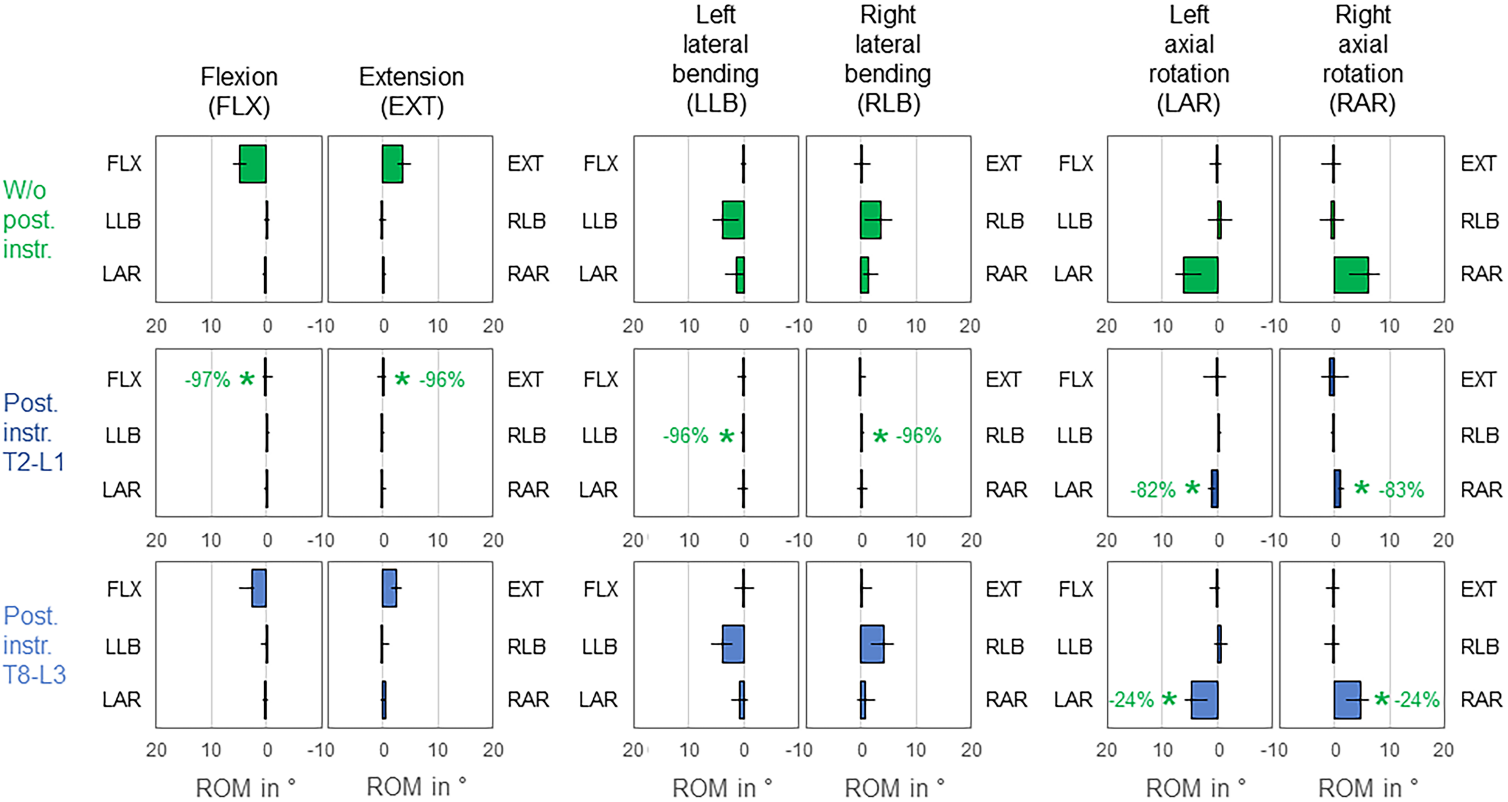
Effects of posterior rod instrumentation on primary and coupled motions of the mid‐thoracic spine (T5‐T9). Data is represented as medians with minimum and maximum values (*n* = 6). Statistical differences (*p* < 0.05) compared to the condition without posterior rod instrumentation are marked with *.

While posterior rod instrumentation did not significantly (*p* > 0.05) alter the coupled motion behavior of the mid‐thoracic spine, T2‐L1 instrumentation almost completely diminished the primary ranges of motion (Figure 7). Highest range of motion reductions were detected in primary flexion (−97%), extension (−96%), and left and right lateral bending (−96%/−96%). T8‐L3 instrumentation significantly (*p* < 0.05) but solely slightly reduced the range of motion in both primary axial rotation directions (−24%/−24%). Coupled motions were overall low in the condition without posterior rod instrumentation and were primarily observed as slight ipsilateral axial rotation during primary lateral bending.

**FIGURE 8:**
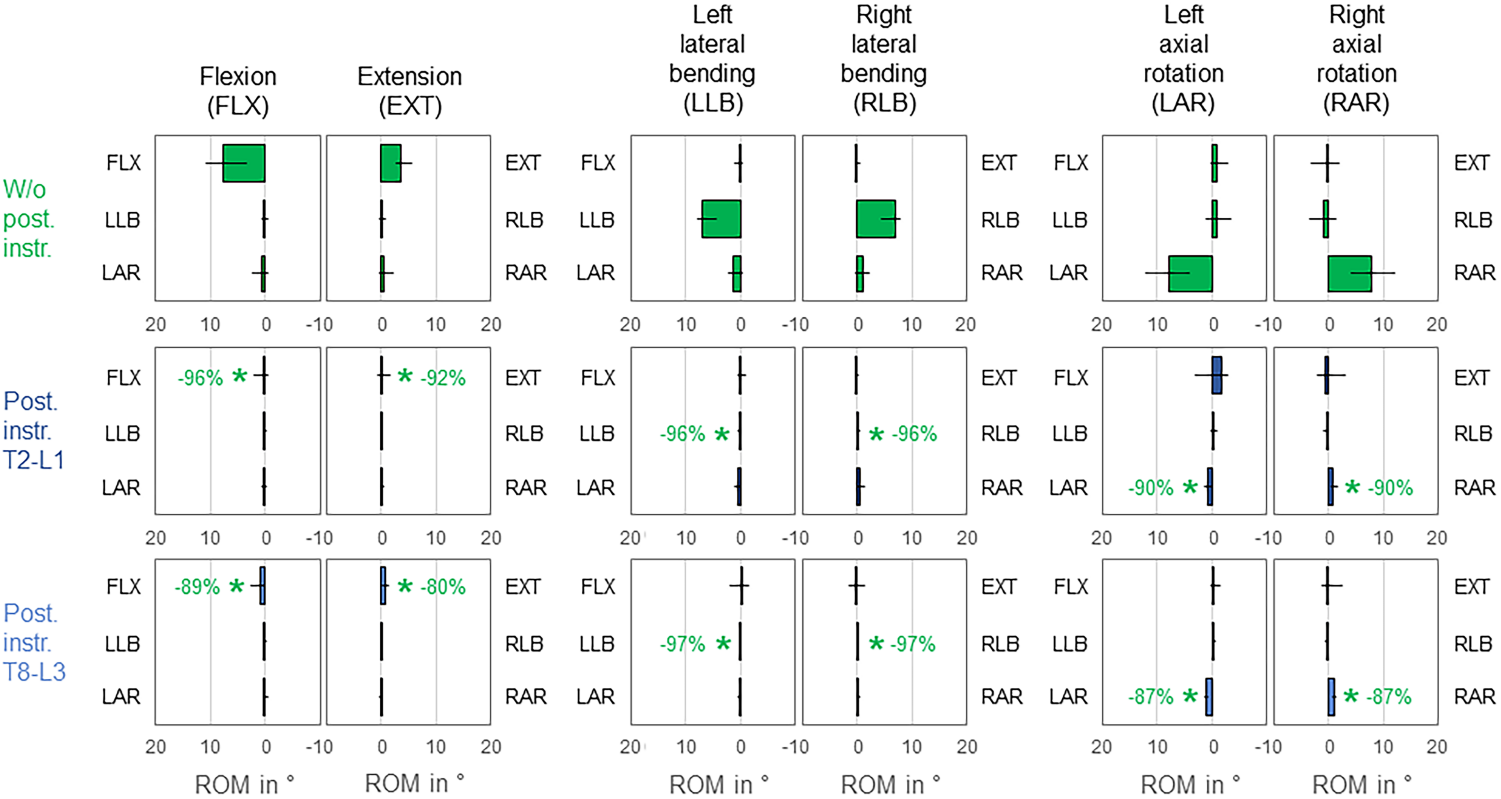
Effects of posterior rod instrumentation on primary and coupled motions of the lower third of the thoracic spine (T9‐L1). Data is represented as medians with minimum and maximum values (*n* = 6). Statistical differences (*p* < 0.05) compared to the condition without posterior rod instrumentation are marked with *.

Both instrumentation types almost equally, completely, and significantly (*p* < 0.05) reduced the primary range of motion of the lower third of the thoracic spine in all 6 motion directions, while the coupled motions remained generally unaffected (Figure 8). Highest reductions of the primary range of motion were found in both directions of lateral bending for both instrumentation types (T2‐L1 instrumentation: −96%/−96%, T8‐L3 instrumentation: −97%/−97%) as well as in flexion for T2‐L1 instrumentation (−96%). Coupled motions were overall low and unspecific in the condition without posterior rod instrumentation.

## 4 DISCUSSION

Post‐operative three‐dimensional correction loss following rigid AIS instrumentation represents a commonly diagnosed complication, while potential influencing factors are still indeterminate. The results of the present study clearly show that common AIS instrumentation configurations not only alter spinal flexibility in the primary motion plane but also restrict the coupled motions of both the thoracic and lumbar spine, indicating a disturbance of the natural three‐dimensional motion behavior and hence potential negative effects on post‐operative correction preservation.

Without posterior rod instrumentation, a strong and reproducible motion coupling between lateral bending and axial rotation was found in the entire thoracic and lumbar spine (T1‐sacrum), while an inverse coupling behavior between thoracic (T1‐L1) and lumbar (L1‐sacrum) spine was detected, which corresponds to the results of a previous in vitro study using whole thoracic and lumbar spine specimens from elderly donors.^16^ More precisely, this inverse motion coupling pattern comprises ipsilateral axial rotation during primary lateral bending and contralateral lateral bending during primary axial rotation in the thoracic spine as well as contralateral axial rotation during primary lateral bending and ipsilateral lateral bending during primary axial rotation in the lumbar spine. The most probable determinant for this phenomenon might be the sagittal plane curvature, more specifically thoracic kyphosis and lumbar lordosis, as already concluded by a computational study.^17^ This might also explain the absence of coupled motions in flexion/extension, since the healthy, non‐deformed spine is almost symmetrical in the frontal plane. Moreover, other factors that have often been discussed as potential causes for coupled motions in the past have been meanwhile widely excluded, as an in vitro study could show that stepwise resection of anatomical structures does not alter the coupled motion behavior of the thoracic spine,^18^ ruling out the facet joints and spinal ligaments as potential determinants for coupled motions. Furthermore, it was shown that the rib cage reduces the absolute thoracic spinal range of motion in both the primary and secondary motion planes,^19^, ^20^, ^21^, ^22^ specifically in case of posterior rod instrumentation,^23^ but does not alter the relative coupled motion behavior.^20^, ^22^, ^24^

Posterior rod instrumentation almost completely eliminated the coupled axial rotation during primary lateral bending in the present study. While it is known that this coupled motion component represents an essential part of the natural three‐dimensional motion behavior,^22^ a previous in vitro study even revealed dominant coupled axial rotation during primary lateral bending in the thoracic spine when being subjected to follower loading for the simulation of body weight and muscle forces.^25^ Diminishing this substantial motion component by rigid spinal fixation therefore represents a major intervention in the natural spinal motion behavior which may result in compensation movements of AIS patients after surgery including excessive loading of the non‐instrumented spinal sections, potentially explaining post‐operative complications such as correction loss and junctional deformity. In particular, coupled motions of the upper thoracic spine were most affected by posterior rod instrumentation from T2 to L1, altering the coupled motions in both primary lateral bending and primary axial rotation and indicating higher risk for complications in the upper thoracic spine. Specifically the thoracic spine was investigated in detail in this study since previous in vitro studies exhibited reproducible coupled motion patterns^16^, ^25^ and their dependency on the length of posterior rod instrumentation in the thoracic spine,^26^ while less motion coupling between lateral bending and axial rotation in the thoracolumbar^27^ and lumbar spine^28^, ^29^ were found in previous investigations. Segmental coupled motions, on the other hand, were not reported in this study, since they were found to be unspecific, non‐reproducible, and mainly detectable among multiple segments in a previous study on the thoracic spine.^25^

The missing testing of scoliotic spinal specimens as a reference condition for the comparison with the two conditions involving posterior rod instrumentation constitutes one limitation of this study. Moreover, scoliosis correction using rigid posterior instrumentation might result in asymmetric load distribution on the rods and the adjacent tissue, which cannot be simulated by using straight spines as in the present study, while the effect on the coupled motion characteristics is not known but probably negligible, as the rigid fixation presumably takes the loads at the instrumented levels in both cases. In general, the coupled motion behavior of scoliotic spines has been solely investigated in vivo so far,^5^, ^6^, ^7^ since specimens from donors with AIS are not accessible due to ethical reasons and usually exhibit heterogeneous deformity characteristics, potentially reducing the reproducibility and interpretability of the results. Therefore, specimens without any clinically relevant deformity from young adult donors were used in order to ensure low variability in primary and coupled motions. Nevertheless, the coupled motion behavior of the scoliotic spine under standardized in vitro conditions remains an important task for future experimental studies. The use of pure moments of 5 Nm for the entire thoracolumbar spinal specimens is a further limitation of the present study, as pure moments do not fully reflect the physiological loading situation and as the moment of 5 Nm is lower than the moment which is usually applied to the lumbar spine for in vitro investigations.^11^ However, pure moments of 5 Nm were chosen as these represent the recommended load for in vitro testing of the thoracic spine^11^ and as the risk of overloading of the thoracic spinal structures should be minimized. Moreover, the additional application of a follower load to simulate the body weight and the muscle forces acting on the spine in upright position was omitted in the present study to keep the overall time of exposure of the specimens to air and room temperature to a minimum, as the appropriate installation of a follower load is complex and time consuming, especially in specimens comprising a large number of segmental levels and with the rib cage present. Yet, the inclusion of a follower load could have led to even higher effects of the instrumentation on the coupled motions, as previous findings determined that follower loading increases the coupled motions of the uninstrumented thoracic spine.^25^ Another limitation of this study represents the investigation of solely two particular instrumentation configurations, while the instrumentation length in terms of the upper and lower instrumented vertebrae was shown to vary considerably depending on the curve type, the severity of the curvature, as well as the individual preference of the surgeon.^13^, ^14^ Although the two selected instrumentations constitute representative configurations, future studies should evaluate effects of other AIS instrumentation lengths on the coupled motion behavior of the thoracic and lumbar spine.

To conclude, the results of this study clearly showed that posterior rod instrumentation for surgical AIS treatment reduces both the primary and the coupled motions of the thoracic and lumbar spine, while instrumentation of the thoracic spine, especially of its upper third, has the highest impact on the coupled motion behavior. In order to prevent three‐dimensional correction loss and junctional deformity and to increase the patients' movement comfort, instrumentations should therefore include as few vertebrae as reasonable with regard to correction stability. Moreover, this study provides reference and validation data for future in vitro, in silico, as well as in vivo studies investigating effects of AIS instrumentation on the three‐dimensional spinal flexibility and motion behavior.

## AUTHOR CONTRIBUTIONS

HJW acquired funding and supervised the research project. CL and BS conceived the study design. CL, MV, and BS developed the methodology. PO and HJW acquired resources. CL, PO, and MV performed the experiments. CL and MV administered, analyzed, and visualized the experimental data. CL wrote the manuscript. PO, MV, BS, and HJW reviewed and edited the manuscript.

## CONFLICT OF INTEREST STATEMENT

The authors declare no conflicts of interest.
